# Tomography patterns of pneumonia caused by various etiologic agents during the first year after kidney transplantation

**DOI:** 10.1590/0100-3984.2021.0069

**Published:** 2022

**Authors:** Luiz Otávio de Andrade Damázio, Esdras Marques Lins, Álvaro Antônio Bandeira Ferraz, Camila de Moraes Bezerra, Fernando Antônio Carneiro Borba Carvalho Neto, Lívia Lócio Rosado de Oliveira, Miguel Calado Soares da Costa, Paula Marina Carneiro Santos

**Affiliations:** 1 Instituto de Medicina Integral Professor Fernando Figueira (IMIP), Recife, PE, Brasil.; 2 Faculdade Pernambucana de Saúde (FPS), Recife, PE, Brasil.; 3 Universidade Federal de Pernambuco (UFPE), Recife, PE, Brasil.

**Keywords:** Pneumonia, Kidney transplantation, Computed tomography, Pneumonia, Transplante de rim, Tomografia computadorizada

## Abstract

**Objective:**

To evaluate the tomography patterns of pneumonia attributed to various etiological agents during the first year after kidney transplantation.

**Materials and Methods:**

We analyzed the medical records of 956 patients who underwent kidney transplantation between 2013 and 2018 at a transplant center in northeastern Brazil. Among the kidney transplant recipients who developed pneumonia, the etiologic agents were categorized as pyogenic bacteria, mycobacteria, fungi, viruses, or polymicrobial pneumonia. The tomography patterns were categorized as consolidation, bronchopneumonia, interstitial pneumonia, or nodules/masses. To determine the statistical association between the causative microorganism and the tomography pattern, we used Fisher’s exact test, for which the level of significance was set at *p* < 0.001.

**Results:**

Among 101 cases of pneumonia reported in kidney transplant recipients, the etiologic agent was identified in 60 (59.4%), the most common category being pyogenic bacteria, which were implicated in 22 cases (36.7%). Among the 60 patients in whom had the causal agent was identified, the pattern in which nodules and masses predominated was the most common, being identified in 25 cases (41.7%). We detected associations between pyogenic bacteria and consolidation, between fungi and nodules/masses, and between viruses and interstitial pneumonia.

**Conclusion:**

There were statistical associations between tomography patterns and the microorganisms that cause pneumonia. This knowledge could facilitate the treatment planning for kidney transplant patients.

## Abstract (pt)

**Objetivo:**

Avaliar os padrões tomográficos relacionados aos agentes etiológicos da pneumonia em pacientes durante o primeiro ano após transplante renal.

**Materiais e Métodos:**

Foram analisados dados de prontuários de 956 pacientes submetidos a transplante renal, no período de 2013 a 2018, em um centro transplantador renal do nordeste do Brasil. Nos pacientes que desenvolveram pneumonia, os agentes etiológicos foram classificados em bactérias piogênicas, micobactérias, fungos, vírus e pneumonia polimicrobiana, enquanto os padrões tomográficos foram classificados em consolidação, broncopneumonia, pneumonia intersticial e nódulos e massas. Para verificar associação estatística entre micro-organismos e padrões tomográficos, foi utilizado o teste exato de Fisher, com *p* < 0,001.

**Resultados:**

Foram encontrados 101 casos de pneumonia, dos quais 60 (59,4%) tiveram agente etiológico identificado, sendo as bactérias piogênicas as mais frequentes, detectadas em 22 (36,7%) dos casos. Entre os pacientes com agente causal identificado, o padrão tomográfico predominante foi o de nódulos e massas, identificado em 25 (41,7%) casos. Foi observada associação entre bactérias piogênicas e o padrão de consolidação, fungos com nódulos e massas, bem como entre agentes virais e padrão intersticial.

**Conclusão:**

Foi demonstrada associação estatística entre micro-organismos causadores de pneumonia e padrões tomográficos, informação que pode contribuir para o planejamento da terapia de pacientes transplantados renais.

## INTRODUCTION

Infection is a major cause of mortality and loss of graft function in kidney transplant recipients, especially during the first year after transplantation, when the immunosuppressive therapy is most intense^(1)^. Pneumonia is one of the main types of such infection, and the severity of pulmonary infection is influenced by the balance of forces between the virulence of the causal agent and the host defenses^(2,3)^.

Diagnostic imaging examinations play a crucial role in the detection of pneumonia, and computed tomography (CT) is a key examination because of its high spatial and temporal resolution, especially when the most modern devices, with multiple rows of detectors, are used^(4)^. Pathogens related to pulmonary infiltrate of an infectious nature can be categorized as viral, bacterial, mycobacterial, or fungal^(5)^, the probability of diagnosis of infection with a specific causal agent being partly determined by the time since transplantation^(6)^, as well as by the socioeconomic status and geographic location of the patient^(7)^. The findings observed on CT can guide antimicrobial therapy for the most likely agents of pneumonia in kidney transplant recipients while cultures are ongoing.

The objective of this study was to evaluate the tomography patterns associated with the etiologic agents of pneumonia in kidney transplant recipients during the first year after transplantation.

## MATERIALS AND METHODS

This was a retrospective, cross-sectional descriptive study, based on the analysis of data related to adult patients undergoing kidney transplantation at the Instituto de Medicina Integral Professor Fernando Figueira (IMIP), in the city of Recife, Brazil, between January 2013 and June 2018. The study was approved by the Research Ethics Committee of the IMIP (Reference no. 03221718.3.0000.5201). Patients who underwent simultaneous transplantation of more than one organ were excluded, as were those who were lost to follow-up, those who died from a cause other than pneumonia during the first year after transplantation, and those for whom the medical records were incomplete.

All CT scans were acquired on one of two scanners: a 64-slice scanner (Brilliance; Philips Medical Systems, Haifa, Israel); and a 6-slice scanner (Somatom Emotion; Siemens Healthcare, Forchheim, Germany).In both CT scanners, the lung parenchyma was studied in high-resolution, slices being acquired from the apices to the bases of the lungs. Thin (1-2 mm) axial slices were obtained with the patient in the supine position, during inspiration, and a high spatial resolution filter was used for image reconstruction.

Both the images and the radiological reports are stored in the hospital’s digital system, being accessed by the researchers in obtaining the study data. The predominant tomography patterns were divided into four categories: consolidation; bronchopneumonia; interstitial pneumonia; and nodules and masses. The criteria for defining those categories were as detailed in the Glossary of Terms created by the Brazilian College of Radiology and Diagnostic Imaging, and Brazilian Society of Pulmonology and Phthisiology^(8)^. In cases in which there was more than one tomography pattern, the predominant pattern was defined as the one that affected the greatest extent of the lung parenchyma.

Within the nodules and masses CT pattern, the findings were categorized as masses when larger than 3.0 cm, as large nodules when between 1.0 cm and 3.0 cm, as small nodules when between 0.3 cm and 1.0 cm, and as miliary nodules when smaller than 0.3 cm.

Samples for microbiological analysis, to identify the causal agent, were obtained by blood collection, sputum collection, bronchoalveolar lavage, transbronchial biopsy, or lung biopsy. The microorganisms were categorized as pyogenic bacteria, viruses, fungi, mycobacteria, or polymicrobial (mixed). In addition to the agent category, the species isolated were also noted. Blood cultures were performed by collecting three samples and analyzing the minimum inhibitory concentrations in an automated system or manually. Peripheral blood samples were sent for cytomegalovirus viral load determination. Before initiation of the specific therapy, samples of spontaneous or induced sputum were collected and sent for Gram and Ziehl-Neelsen staining. If appropriate, they were sent to by cultured for bacteria, *Mycobacterium tuberculosis*, and fungi. Bronchoalveolar lavage fluid samples were sent for direct slide analysis, cell counts, culture for *Nocardia* sp., polymerase chain reaction for detection of *M. tuberculosis*, and fungal culture. Specimens collected via transbronchial biopsy or via lung biopsy performed under direct vision were processed using routine techniques and sent to the pathology laboratory.

Statistical analyses were performed with the Stata statistical software package, version 11 (StataCorp, College Station, TX, USA). To determine whether there was an association between a given etiologic agent and a given tomography pattern, we used Fisher’s exact test. The significance threshold adopted was *p* < 0.001.

## RESULTS

We analyzed the medical records of 965 patients who underwent kidney transplantation at the IMIP between January 2013 and June 2018. Among those patients, there were 101 cases of pneumonia after transplantation.

Among the 101 patients with pneumonia (Table 1), the microbiological agent was identified in 60 (59.4%), the most common category being pyogenic bacteria, which were implicated in 22 (36.7%) of those 60 cases, followed by mycobacteria, in 13 (21.6%), fungi, in eight (13.3%), and viruses, in seven (11.6%). In the remaining ten cases (16.7%), more than one causal agent was identified (Table 2), and those were therefore classified as cases of polymicrobial pneumonia.

**Table 1:** Characteristics and microbiological profile of patients who developed pneumonia in the first year after kidney transplantation.

Variable	(n = 101)
Death, n	15
Mean age (years)	46.0
Gender, n	
Male	67
Female	34
Post-transplant period in which pneumonia developed, n	
Early (< 30 days)	5
Intermediate (30-180 days)	75
Late (> 180 days)	21
Etiologic agent identified, n	60
Etiologic agent class, n	
Bacterial	22
Mycobacterial	13
Viral	7
Fungal	8
Polymicrobial (mixed)	10

**Table 2:** Absolute numbers of pneumonia cases, by causal agent.

Etiologic agents	(n = 60)
Pyogenic bacteria	
*Acinetobacter baumannii*	3
*Burkholderia cepacia*	1
*Escherichia coli*	3
*Klebsiella pneumoniae*	7
*Pseudomonas aeruginosa*	7
*Serratia marcescens*	1
Mycobacteria	
*Mycobacterium tuberculosis*	13
Polymicrobial (mixed)	
*Mycobacterium tuberculosis + Klebsiella pneumoniae*	2
*Acinetobacter baumannii + Pseudomonas aeruginosa*	2
*Staphylococcus aureus + Mycobacterium tuberculosis*	1
*Mycobacterium tuberculosis + Aspergillus flavus*	1
*Klebsiella pneumoniae + Candida* sp.	1
*Escherichia coli + Candida albicans*	1
*Klebsiella pneumoniae + Pseudomonas aeruginosa + Serratia marcescens*	1
*Klebsiella pneumoniae + Candida* sp. *+ Aspergillus nidulans*	1
Fungi	
*Aspergillus flavus*	1
*Candida* sp.	1
*Candida albicans*	1
*Cryptococcus neoformans*	4
*Histoplasma* sp.	1
Virus	
Cytomegalovirus	7

**Figure 1:**
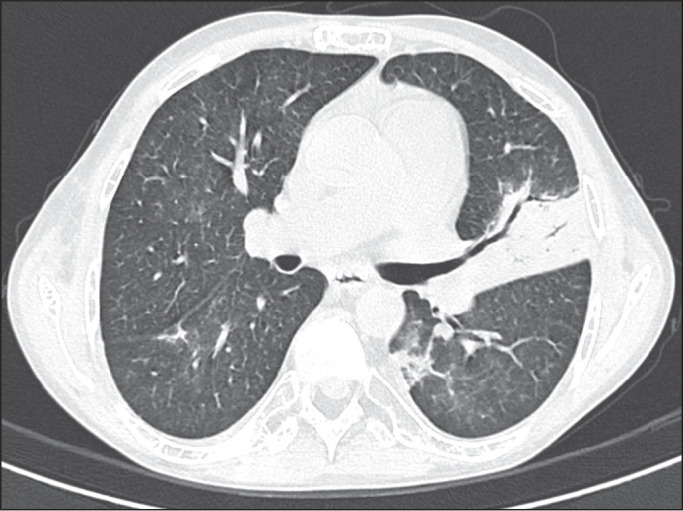
A 34-year-old man who developed cough and fever in the fifth month post-transplant. CT scan showing parenchymal consolidation in the lingula. Note the discrete ground-glass opacities in the lower lung lobes and the small focus of consolidation in the left lower lobe. Blood culture revealed *A. baumannii*.

**Figure 2:**
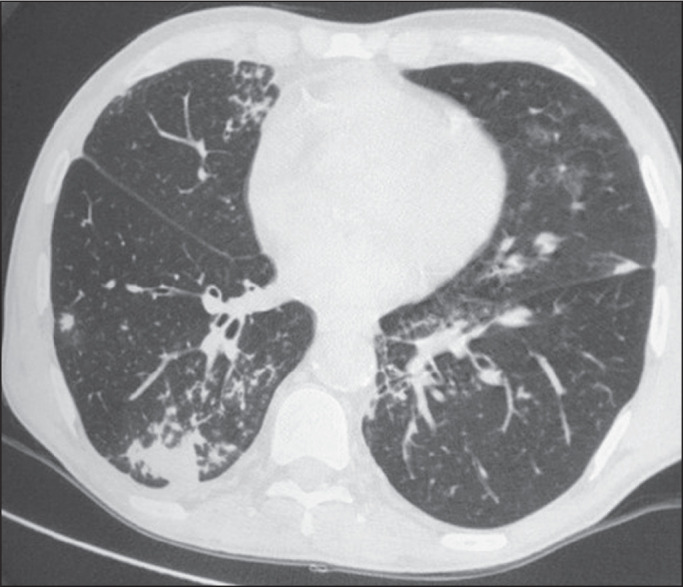
A 60-year-old man who presented with a 15-day history of a productive cough in the tenth month post-transplantation. CT scan showing a bronchopneumonia pattern with multifocal centrilobular nodules beginning to coalesce, forming small foci of consolidation, the largest in the right lower lobe. There is also thickening of the bronchial walls and a few sparsely distributed airspace nodules. Examination of the bronchoalveolar lavage fluid revealed *M. tuberculosis*.

**Figure 3:**
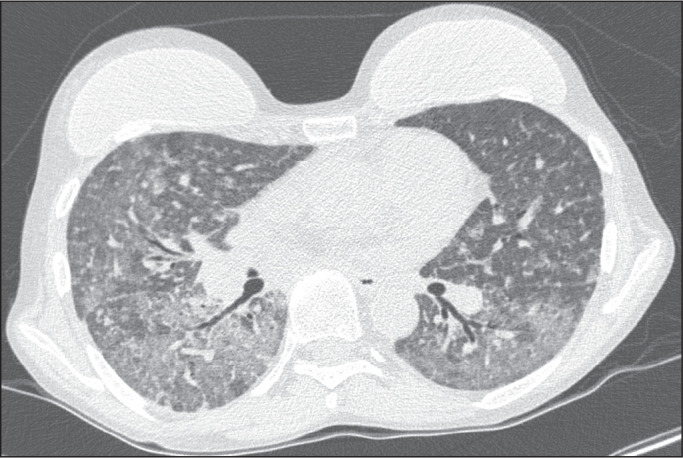
A 40-year-old woman with fever, dyspnea and hypoxemia in the second month post-transplant. CT scan showing an interstitial pattern with diffuse ground-glass opacities. Antigenemia for cytomegalovirus was positive.

**Figure 4:**
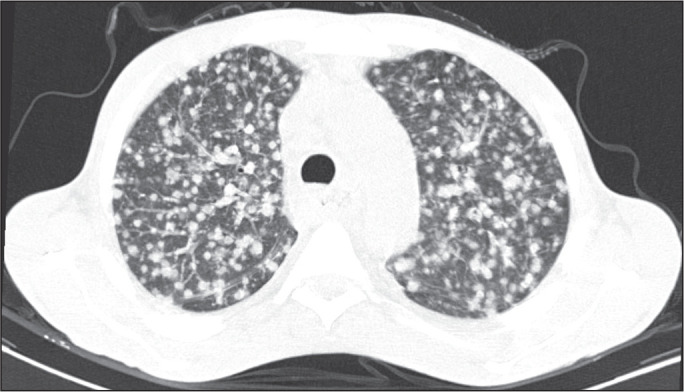
A 34-year-old man with disseminated candidiasis in the fourth month post-transplant. CT scan showing multiple pulmonary nodules.

**Figure 5:**
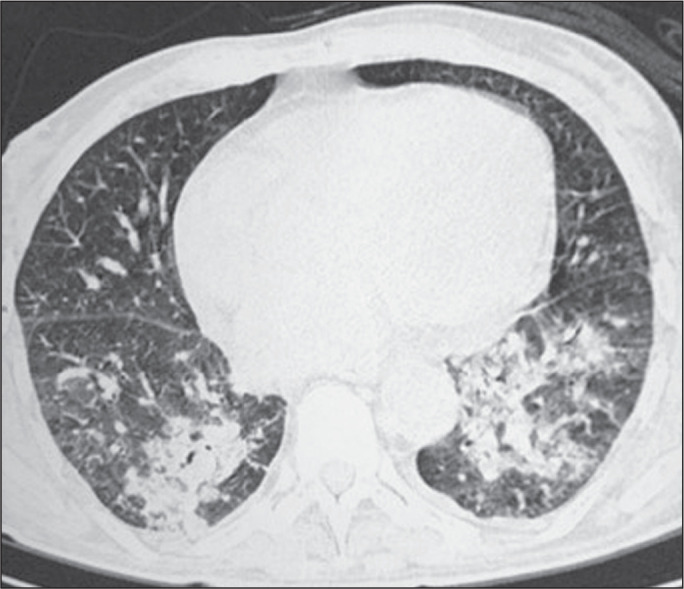
A 70-year-old man with a 7-day history of fever, dyspnea, and productive cough in the second month post-transplantation. CT scan showing airspace consolidations in both lower lobes. The laboratory investigation of the etiologic agent was inconclusive.

Among the 60 patients who had the causal agent identified, the nodules and masses tomography pattern was the only or predominant pattern in 25 (41.7%), whereas the predominant pattern was bronchopneumonia in 16 (26.7%), consolidation in 13 (21.7%), and interstitial pneumonia in six (10.0%). Examples of cases in which the consolidation, bronchopneumonia, interstitial pneumonia, and nodules/masses tomography patterns were observed are shown in Figures 1, 2, 3, and 4, respectively. Figure 5 shows a CT scan of a patient with pneumonia in which the nodules/masses tomography pattern was observed and it was not possible to isolate the causal agent. The distribution of cases in which the nodules/masses tomography pattern was observed is shown in Table 3. The associations between the classes of etiological agents and the tomography patterns, as assessed with Fisher’s exact test, are shown in Table 4.

**Table 3:** Distribution of the cases of pneumonia in which the nodules and masses tomography pattern was observed, by lesion size.

Category	(n = 25)
Masses (> 3.0 cm), n (%)	5 (15.7)
Large nodules (1.0-3.0 cm), n (%)	10 (31.2)
Small nodules (0.3-1.0 cm), n (%)	10 (31.2)
Miliary nodules (< 0.3 cm), n (%)	7 (21.9)

**Table 4:** Fisher’s test results.

Etiologic agents	Consolidation	Bronchopneumonia	Interstitial	Nodules and masses
Pyogenic bacteria	(n = 8) Absolute value: 4.767 Adjusted residual: 2.103	(n = 8) Absolute value: 5.867 Adjusted residual: 1.292	(n = 0) Absolute value: 2.200 Adjusted residual: -1.965	(n = 6) Absolute value: 9.167 Adjusted residual: -1.721
Polymicrobial (mixed)	(n = 3) Absolute value: 2.167 Adjusted residual: 0.701	(n = 2) Absolute value: 2.667 Adjusted residual: -0.522	(n = 0) Absolute value: 1.000 Adjusted residual: -1.155	(n = 5) Absolute value: 4.167 Adjusted residual: 0.586
Fungi	(n = 0) Absolute value: 1.733 Adjusted residual: -1.598	(n = 0) Absolute value: 2.133 Adjusted residual: -1.832	(n = 0) Absolute value: 0.800 Adjusted residual: -1.013	(n = 8) Absolute value: 3.333 Adjusted residual: 3.595
Viruses	(n = 0) Absolute value: 1.517 Adjusted residual: -1.481	(n = 1) Absolute value: 1.867 Adjusted residual: -0.788	(n = 6) Absolute value: 0.700 Adjusted residual: 7.105	(n = 0) Absolute value: 2.917 Adjusted residual: -2.379
Mycobacteria	(n = 2) Absolute value: 2.817 Adjusted residual: -0.621	(n = 5) Absolute value: 3.467 Adjusted residual: 1.087	(n = 0) Absolute value: 1.300 Adjusted residual: -1.358	(n = 6) Absolute value: 5.417 Adjusted residual: 0.371

## DISCUSSION

The assessment of pulmonary infections by imaging methods, particularly CT, has been the subject of a number of recent studies in the radiology literature of Brazil^(9-13)^. According to data in the international literature, the consolidation pattern is often associated with pneumonia caused by pyogenic bacteria^(7)^. In a study involving 114 cases of pneumonia, 35 of which were in immunosuppressed patients, Reittner et al.^(14)^ found consolidation to be the most common alteration, occurring in 85% of the cases. In a study with bone marrow transplant recipients with bacterial pneumonia, Coelho et al.^(15)^ reported that consolidation occurred in 60% of cases. In the present study, similar to what is seen the literature, pyogenic bacteria were responsible for approximately 60% of the cases of pneumonia with the consolidation pattern.

Among cases with the bronchopneumonia tomography pattern, which is the most prevalent pattern in patients with hospital-acquired pneumonia, atypical bacteria constitute one of the most common causes^(14)^. On CT, the presence of centrilobular nodules is indicative of bronchiolar inflammation, which is the main finding in the bronchopneumonia pattern. In the present study, pyogenic bacteria were the main etiologic agent of bronchopneumonia, accounting for half of the cases in which the etiologic agent was identified, although there was no statistically significant association between the etiologic agent class and bronchopneumonia.

The interstitial pneumonia pattern results from inflammation and edema that occur primarily in the pulmonary interstitium, resulting from the aggression caused by an infectious agent, usually a virus, cytomegalovirus being the most prevalent causative agent after kidney transplantation^(16)^. Among the patients in whom the etiologic agent was identified in our study, there were six cases with an interstitial pattern, all of which were attributed to cytomegalovirus infection. Recently, severe acute respiratory syndrome coronavirus 2, initially identified in the city of Wuhan, China, in December 2019, has also caused pneumonia with an interstitial pattern in transplant recipients, although there have still been few studies of that scenario^(17)^.

Pulmonary nodules are common findings in immunosuppressed patients with pulmonary infection. Copp et al.^(18)^ reported that nodules of an infectious nature were found in 56% of patients who underwent solid organ transplantation and subsequently developed pneumonia. In a study involving kidney transplant recipients only, Gandhi et al.^(19)^ observed a nodular pattern (excluding centrilobular nodules) in 25% of the cases and miliary mottling in 6%, as well as a slight predominance of fungal diseases. In our sample, the nodular pattern was the most common tomography pattern, observed in approximately 40% of the cases in which the etiologic agent was identified. Fungi were the most common etiologic agents, accounting for 32% of the cases with a nodular pattern. It is possible that the predominance of the nodules and masses pattern was due to the fact that the investigation of the causal agent was more in-depth in our sample, involving specific diagnostic tests, given that only patients with a defined etiological agent were evaluated. It is likely that if all patients were evaluated, including those with pneumonia caused by an undefined microorganism, there would be a predominance of the consolidation pattern. Many of the patients responded to empirical treatment and therefore did not undergo etiological investigation.

In a study evaluating aspects of the nodular tomography pattern in immunosuppressed patients with nodules of an infectious nature, Franquet et al.^(20)^ reported that at least one nodule or mass larger than 1.0 cm was found in 64 (82.1%) of the 78 patients in the sample as a whole and in 19 (86.4%) of the 22 patients with fungal infection. According to Torres et al.^(21)^, nodules are often seen in patients with pulmonary mycoses, which can be severe in patients who are immunocompromised, in whom pulmonary involvement remains the most common documented form of invasive infection of tissue.

In the present study, Fisher’s test revealed statistical associations that were expected on the basis of the literature^(22,23)^: between the consolidation pattern and infection with pyogenic bacteria; between the nodules/masses pattern and fungal infection; and between the interstitial pneumonia pattern and viral infection. However, there have been few studies involving multivariate analysis in kidney transplant recipients. One such study, conducted by Jiang et al.^(24)^, had the same objective as the present study but considered a greater variety of imaging aspects, using subdivisions of the patterns established in our study. That could explain why those authors did not find any statistically significant associations between the variables, as were found in our study.

Our study has some limitations. The retrospective design made the study more susceptible to biases, and the small number of cases with a defined causal agent limits the power of the results. Nevertheless, it established the incidence and mortality of pneumonia during the first year after kidney transplantation at the largest kidney transplant center in the northern/northeastern region of Brazil and demonstrated a statistical association between microorganisms causing pneumonia and tomography patterns, thus contributing to the planning of the treatment of kidney transplant patients.

## CONCLUSION

In our sample, the most common tomography pattern was that of nodules and masses. There was a higher-than-expected frequency of the consolidation tomography pattern, which showed a statistical association with bacterial pneumonia, as well as a higher-than-expected frequency of the interstitial pneumonia pattern, which showed a statistical association with viral pneumonia. There was also a stronger-than-expected statistical association between fungal pneumonia and the nodules/masses tomography pattern.

The CT patterns found in pneumonia during the first year after kidney transplantation are potentially useful for the differential diagnosis among infections with the various types of etiologic agents. Our findings could facilitate the early treatment of patients with such infections.
